# Impaired Endothelial Progenitor Cell Mobilization and Dysfunctional Bone Marrow Stroma in Diabetes Mellitus

**DOI:** 10.1371/journal.pone.0060357

**Published:** 2013-03-28

**Authors:** Peter E. Westerweel, Martin Teraa, Shahin Rafii, Janneke E. Jaspers, Ian A. White, Andrea T. Hooper, Pieter A. Doevendans, Marianne C. Verhaar

**Affiliations:** 1 Department of Nephrology and Hypertension, University Medical Center Utrecht, Utrecht, The Netherlands; 2 Howard Hughes Medical Institute, The Ansary Stem Cell Center for Regenerative Medicine, Weill Cornell Medical College, New York, New York, United States of America; 3 Department of Haematology, University Medical Center Utrecht, Utrecht, The Netherlands; 4 Department of Vascular Surgery, University Medical Center Utrecht, Utrecht, The Netherlands; 5 Department of Cardiology, University Medical Center Utrecht, Utrecht, The Netherlands; University of Padova, Medical School, Italy

## Abstract

**Background:**

Circulating Endothelial Progenitor Cell (EPC) levels are reduced in diabetes mellitus. This may be a consequence of impaired mobilization of EPC from the bone marrow. We hypothesized that under diabetic conditions, mobilization of EPC from the bone marrow to the circulation is impaired –at least partly– due to dysfunction of the bone marrow stromal compartment.

**Methods:**

Diabetes was induced in mice by streptozotocin injection. Circulating Sca-1^+^Flk-1^+^ EPC were characterized and quantified by flow cytometry at baseline and after mobilization with G-CSF/SCF injections. *In vivo* hemangiogenic recovery was tested by 5-FU challenge. Interaction within the bone marrow environment between CD34^+^ hematopoietic progenitor cells (HPC) and supporting stroma was assessed by co-cultures. To study progenitor cell–endothelial cell interaction under normoglycemic and hyperglycemic conditions, a co-culture model using E4Orf1-transfected human endothelial cells was employed.

**Results:**

In diabetic mice, bone marrow EPC levels were unaffected. However, circulating EPC levels in blood were lower at baseline and mobilization was attenuated. Diabetic mice failed to recover and repopulate from 5-FU injection. *In vitro*, primary cultured bone marrow stroma from diabetic mice was impaired in its capacity to support human CFU-forming HPC. Finally, hyperglycemia hampered the HPC supportive function of endothelial cells *in vitro*.

**Conclusion:**

EPC mobilization is impaired under experimental diabetic conditions and our data suggest that diabetes induces alterations in the progenitor cell supportive capacity of the bone marrow stroma, which could be partially responsible for the attenuated EPC mobilization and reduced EPC levels observed in diabetic patients.

## Introduction

Premature atherosclerosis is a major complication in diabetes [1], which is at least in part attributable to an impaired vascular regenerative potential of bone marrow-derived progenitor cells [2]. Diabetes is associated with endothelial cell dysfunction and impaired neovascularization after ischemia [3]–[6]. Healthy intact endothelium and maintenance of its integrity play a central role in protecting against the development of atherosclerotic disease [7]. Endothelial progenitor cells (EPC), a specialized subset of hematopoietic progenitor cells (HPC), circulate in peripheral blood and contribute to restoring damaged or lost endothelium and facilitate ischemic neovascularization [8]. EPC are capable of endothelial differentiation [9] and secretion of angiogenic growth factors and cytokines [10], [11], which is of paramount importance in neovascularization. Similar to HPC, EPC originate from the bone marrow, from which they are mobilized in response to mobilizing cytokines, such as vascular endothelial growth factor (VEGF) [12], granulocyte colony-stimulating factor (G-CSF) [13], [14], and stromal-derived factor-1α (SDF-1α) [15], and neuronal impulses [16], [17]. In the bone marrow HPC are localized in two distinguishable stem cell niches, i.e. the ‘osteoblastic’ and the ‘vascular niche’, which largely regulate progenitor cell proliferation and mobilization. Progenitor cell proliferation and quiescence is thought to be predominantly regulated by osteoblasts in the ‘osteoblastic niche’, which is further composed of a heterogeneous population of stromal cells that includes fibroblasts and endothelial cells. The sinusoidal endothelium is the essential component of the ‘vascular niche’ and plays a pivotal role in progenitor cell egress from the bone marrow to the circulation [18]–[20], a process that requires nitric oxide (NO) [21].

Previous studies showed reduced HPC and EPC levels in type 1 and 2 diabetic patients [22]–[24]. Low levels of circulating EPC in peripheral blood may undermine the regenerative potential of the endothelium and thus contribute to accelerated cardiovascular disease development. Indeed, in prospective cohort studies, lower levels of EPC were associated with poor event-free survival [25], [26]. Interestingly, endothelial dysfunction, measured by flow-mediated brachial artery reactivity, correlates with reduced EPC numbers in the peripheral circulation of patients at risk for cardiovascular disease [27]. This suggests that endothelial dysfunction and reduced EPC levels share a common pathogenic mechanism. Although circulating levels of EPC may also be reduced due to increased endothelial turnover or decreased cell survival, recent experimental evidence in diabetic murine models supports a role for impaired mobilization and a deranged bone marrow microenvironment [6], [28]–[30] and bone marrow innervation [31]. We hypothesized that under diabetic conditions, endothelial progenitor cell levels are reduced due to impaired progenitor cell mobilization from the bone marrow in association with dysfunction of the bone marrow stroma.

## Methods

### Animal model of diabetes

Diabetes was induced in 6-week-old C57Bl/6 mice (*n* = 9; Harlan Laboratories Inc, Indianapolis, IN, USA) by a single intraperitoneal injection of 200 mg/kg streptozotocin (STZ, Serva Feinbiochemica GMBH, Heidelberg, Germany) in citrate buffer (pH 4.5), as published previously [32]. Buffer injected littermates served as controls (*n* = 10). Diabetic mice received suboptimal insulin treatment by subcutaneous implantation of an insulin-releasing pellet (Linbit, Linshin Canada Inc, Toronto, Ontario, Canada) to prevent severe catabolism and lethal diabetes. Diabetic animals were required to have non-fasting blood glucose levels of >15 mmol/l after insulin pellet placement to be included in the study. Blood glucose levels were measured with a portable glucose meter (Medisense, Abbot Laboratories, Abbott Park, IL, USA). Further experiments were performed 4 weeks after confirmation of hyperglycemia. All experiments were performed at the same time of the day and in coupled-pairs of diabetic and non-diabetic animals to circumvent potential circadian influences on progenitor cell numbers.

### Ethics statement

All protocols of animal experiments were approved by the animal ethical committee of the University Medical Center Utrecht, The Netherlands, or the Weill Cornell Medical Center, New York, USA.

Protocols with respect to isolation of cells and cell lines were approved by the institutional review board of the University Medical Center Utrecht, The Netherlands, or the Weill Cornell Medical Center, New York, USA. All subjects provided written informed consent.

### Quantification of HPC and EPC in blood and bone marrow

Flow cytometric quantification of both Sca-1^+^ and c-Kit^+^ HPC and EPC (Sca-1^+^Flk-1^+^) was performed in peripheral blood and bone marrow. On different time points EDTA anticoagulated blood was obtained via femoral vein cannulation and bone marrow cell suspension by flushing the femurs of the mice with RPMI medium (Invitrogen Ltd, Carlsbad, CA, USA) during the terminal experiment. Subsequently, 50 µl of whole blood or a volume of bone marrow suspension containing 1×10^6^ cells was stained with α-Sca1-FITC (BD Pharmingen, San Diego, CA, USA) and α-Flk1-PE (BD Pharmingen) or α-cKit-FITC (BD Pharmingen). Erythrocytes were lysed in an ammonium chloride buffer and remaining cells analyzed with flow cytometry (FC 500, Beckman Coulter, Fullerton, CA, USA). Cell numbers were quantified per ml of blood, estimated based on their relative proportion to the leucocytes in the flow cytometry sample and the total number of leucocytes in a complete blood cell count made on a hematocytometer (Cell-dyn 1800, Abbott laboratories), and for bone marrow cell numbers per femur were estimated based on their relative proportion in the sample and the total number of isolated cells. Additionally, progenitor cells were also quantified as percentage of the total number of WBC in either blood or bone marrow.

From bone marrow cell suspensions (diabetic, *n* = 4; control, *n* = 4; three experiments per mouse), an additional EPC quantification was performed by culturing bone marrow cells at a density of 2×10^6^ cells/well on fibronectin-coated cover slips in 24-wells plates in EGM-medium supplemented with 20% FCS, 100 ng/ml VEGF (R&D Systems Inc, Minneapolis, MN, USA) and penicillin/streptomycin (Invitrogen Ltd). After 4 days, non-adherent cells were washed away and adherent cells were incubated with DiI-labeled acetylated-LDL (ac-LDL, Invitrogen Ltd), fixed in paraformaldehyde, stained with FITC-labeled BS1-lectin (Bioconnect B.V., Huissen, The Netherlands), DAPI, and then mounted on slides using Vectashield (Vector Labs, Burlingame, CA, USA). EPC were identified as ac-LDL/lectin double-positive cells using a fluorescence microscope and quantified using the average cell count in 3 random high-power fields.

### Assessment of progenitor cell mobilization

HPC and EPC mobilization was assessed after subcutaneous injections of 250 µg/kg granulocyte-colony stimulating factor (G-CSF, Neupogen®, Amgen, Thousand Oaks, CA, USA) and 50 µg/kg stem cell factor (SCF, Amgen) in 0,9% NaCl for five consecutive days (experimental days 0 to 4), as previously published [33]. HPC and EPC in peripheral blood were quantified at baseline (prior to G-CSF and SCF injections) and at experimental days 2, 4, 7 and 10 after induction of progenitor cell mobilization.

### *In vivo* model for vascular niche function: 5-Fluorouracil challenge

Control and diabetic mice were intravenously injected with 250 mg/kg body weight 5-fluorouracil (5-FU; American Pharmaceutical Partners, Schaumburg, IL, USA). White blood cell (WBC) and platelet counts were monitored in peripheral blood samples using a hematocytometer at baseline and after 4, 7, 10, 14 and 21 days.

### Isolation of human CD34^+^ hematopoietic progenitor cells

Human CD34^+^ HPC were isolated from umbilical cord blood (CB) by magnetic activated cell sorting (MACS) using a commercially available CD34^+^ isolation kit (Miltenyi Biotech, Auburn, CA, USA) according to the manufacturer's instructions. In brief, mononuclear cells were isolated using Ficoll-density gradient separation (Amersham Biosciences, Piscataway, NJ, USA) and incubated with magnetic microbead-conjugated α-CD34-antibodies and FcR-blocking solution. Cells were passed over a selection column (LS column, Miltenyi Biotech) placed in a magnetic field. After removal of the column from the magnetic field, positive cells were eluded and the procedure was repeated using a second column. Purity of selected CD34^+^ cells was evaluated with flow cytometry using α-CD34-FITC (BD Pharmingen). Mean purity was 91% (range 71–96%) in the isolations performed for the experiments in this study. To prevent potential effects of differences in CD34^+^ purity coupled diabetic and non-diabetic experiments using the same progenitor cell sample were performed throughout this study.

### *Ex vivo* model for bone marrow stroma – progenitor cell interaction

Primary mouse bone marrow stromal cells (BMSC) were obtained by isolating the plastic-adherent fraction from crude bone marrow cell suspensions. Bone marrow cells were flushed from mouse femurs using RPMI medium and cultured in DMEM (Invitrogen Ltd) containing 20% FCS and penicillin/streptomycin (Invitrogen Ltd) at a density of 1×10^7^ cells per T25 culture flask. Medium was changed after one week and subsequently every 2–3 days until cells reached confluence. Subsequently, mouse BMSC were trypsinized and passed into a 12-wells plate and co-cultured with 1×10^5^ human cord blood CD34^+^ HPC (CB-HPC) in X-VIVO-20 medium (Biowhittaker Inc, Chesterbrook, PA, USA) containing 2% FCS. After 10 days, non-adherent and trypsinized adherent cells were pooled and a fraction was plated in methylcellulose medium containing hematopoietic growth factors (Methocult complete, StemCell Technologies, Vancouver, BC, Canada). The number of colony forming units (CFU) was quantified after 14 days of culture (diabetic, *n* = 8; control, *n* = 10).

### *In vitro* model for the bone marrow vascular niche

Human umbilical vein endothelial cells (HUVEC) were isolated as previously described [34] and transfected with a lentiviral vector to express the E4Orf1 construct, providing endothelial cells with the capacity for long term support of hematopoietic cells in a confluent state as recently described [35]. E4Orf1-transfected HUVEC were grown to confluence in 12-wells plates, after which 1×10^5^ CD34^+^ CB-HPC per well were added to the culture. Co-cultures were maintained in IMDM medium (Invitrogen Ltd) containing 0 or 30 mM added D-Glucose (Sigma Aldrich, St. Louis, MO, USA). A small volume of fresh medium was added every 2–3 days and every two weeks excessive medium was carefully removed with minimal aspiration of non-adherent cells. Glucose concentrations were carefully monitored throughout the experiments to verify the normo- and hyperglycemic culture conditions. Glucose concentrations oscillated between 3–8 mM for the normoglycemic experiments and were approximately 30 mM in hyperglycemic cultures. After 2, 4 and 6 weeks, the number of non-adherent cells was counted and then again pooled with the adherent co-cultured cells, which were detached using trypsin-EDTA (Invitrogen Ltd). A fraction of these cells was plated in methylcellulose medium containing hematopoietic growth factors (Methocult complete, StemCell Technologies) and evaluated for generation of CFU after 14 days culture.

### Assessment of direct effects of hyperglycemia on CD34^+^ CB-HPC survival and migratory function

CD34^+^ CB-HPC were incubated overnight in IMDM medium containing 10% FCS and 0 or 30 mM added D-Glucose, or 30 mM D-Mannitol (Sigma Aldrich) as osmotic control. Cell number was assessed using a Bürker-Türk counting chamber, counting only viable cells based on Trypan-Blue exclusion. Equal cell numbers were taken up in IMDM medium containing 1% FSC and 0 or 30 mM added D-Glucose, or 30 mM D-Mannitol and placed in transwell insert (5μm pore size, Corning Costar, Cambridge, MA, USA) in a 24-well migration system with 100 ng/ml SDF-1α (R&D Systems) or vehicle added to the bottom wells. After 4 hours, migrated cells were counted using an automated cell counter (Cell-dyn 1800, Abbott Laboratories).

### Statistical analysis

Data are expressed as means±SEM and were analyzed using SPSS version 20.0 (SPSS Inc, Chicago, IL, USA). After testing for normal distribution of data and equality of variances, differences between groups were analyzed using an unpaired-samples Student's *t*-test. In time elapsing experiments differences within groups compared to baseline were analyzed using repeated measures ANOVA with Bonferroni post-hoc testing. Between group differences in experiments with different time points were analyzed with a two-way ANOVA with Bonferroni post-hoc testing. A P-value of <0.05 was considered statistically significant.

## Results

### Diabetic mice have normal bone marrow EPC levels, but EPC mobilization is impaired

The number of Sca-1^+^Flk-1^+^ EPC isolated per femur did not differ between diabetic and control mice (diabetic, 13.2±3.7; control, 10.5±3.4×10^3^; P = 0.591). A similar picture was observed for both Sca-1^+^ (diabetic, 4.0±0.6; control, 3.4±0.6×10^6^; P = 0.449) and c-Kit^+^ (diabetic, 1.8±0.2; control, 1.5±0.2×10^6^; P = 0.297) HPC bone marrow content (Table 1). Consistently, quantification of EPC by ex vivo culture of bone marrow cells also revealed no significant differences in cell number (diabetic, 24.1±2.2; control, 24.4±5.2 per high power field; P = 0.965).

**Table 1:** Progenitor cell numbers in blood and bone marrow.

		Sca-1^+^		Sca-1^+^Flk-1^+^ EPC		c-Kit^+^	
		DM	Control	DM	Control	DM	Control
Absolute numbers							
PB (/ml)	Baseline	2.3±0.3×10^6, ##^	4.1±0.3×10^6^	7.7±0.9×10^3, #^	13.3±1.9×10^3^	0.12±0.03×10^6^	0.15±0.02×10^6^
	t = 2	3.7±0.4×10^6, ##^	7.5±0.9×10^6, *^	13.0±1.4×10^3, ##^	29.9±5.0×10^3, *^	0.38±0.03×10^6, ##,**^	0.61±0.07×10^6, **^
	t = 4	3.9±0.5×10^6, ##,^ *	9.4±0.6×10^6, **^	12.0±1.3×10^3, ##, *^	30.5±3.2×10^3, **^	0.44±0.06×10^6, ##, *^	0.74±0.06×10^6, **^
	t = 7	2.2±0.2×10^6, ##^	4.7±0.3×10^6^	12.7±5.7×10^3^	15.5±2.2×10^3^	0.20±0.05×10^6^	0.28±0.06×10^6^
	t = 10	2.4±0.3×10^6, #^	3.7±0.4×10^6^	11.8±2.2×10^3^	13.5±2.3×10^3^	0.19±0.03×10^6^	0.11±0.02×10^6^
BM (/femur)		4.0±0.6×10^6^	3.4±0.6×10^6^	13.2±3.7×10^3^	10.5±3.4×10^3^	1.8±0.2×10^6^	1.5±0.2×10^6^
% Cells relative to WBC numbers							
PB	Baseline	5.1±0.2 ^##^	6.7±0.1	0.18±0.01	0.21±0.02	2.3±0.5	2.5±0.2
	t = 2	4.2±0.2 ^**^	4.6±0.3 ^**^	0.15±0.01	0.17±0.02	4.5±0.1 ^*^	3.7±0.2 ^*^
	t = 4	4.3±0.2 ^#^	3.5±0.2 ^**^	0.13±0.01 ^**^	0.12±0.01 ^**^	5.2±0.7 ^#^	2.8±0.2
	t = 7	5.3±0.2 ^##^	6.9±0.1	0.17±0.01	0.22±0.02	5.0±1.0	3.9±0.7
	t = 10	4.1±0.3 ^##, **^	6.4±0.2	0.20±0.03	0.22±0.02	3.4±0.5 ^#^	1.9±0.2
BM		7.8±0.7	7.5±0.5	0.03±0.01	0.02±0.01	3.6±0.3	3.8±0.4

DM  =  Diabetes Mellitus, PB  =  Peripheral Blood, BM  =  Bone Marrow, WBC  =  White Blood Cell. ^#^ P<0.05 compared to controls, ^##^ P<0.01 compared to controls, * P<0.05 compared to baseline, ** P<0.01 compared to baseline.

**Figure 1:**
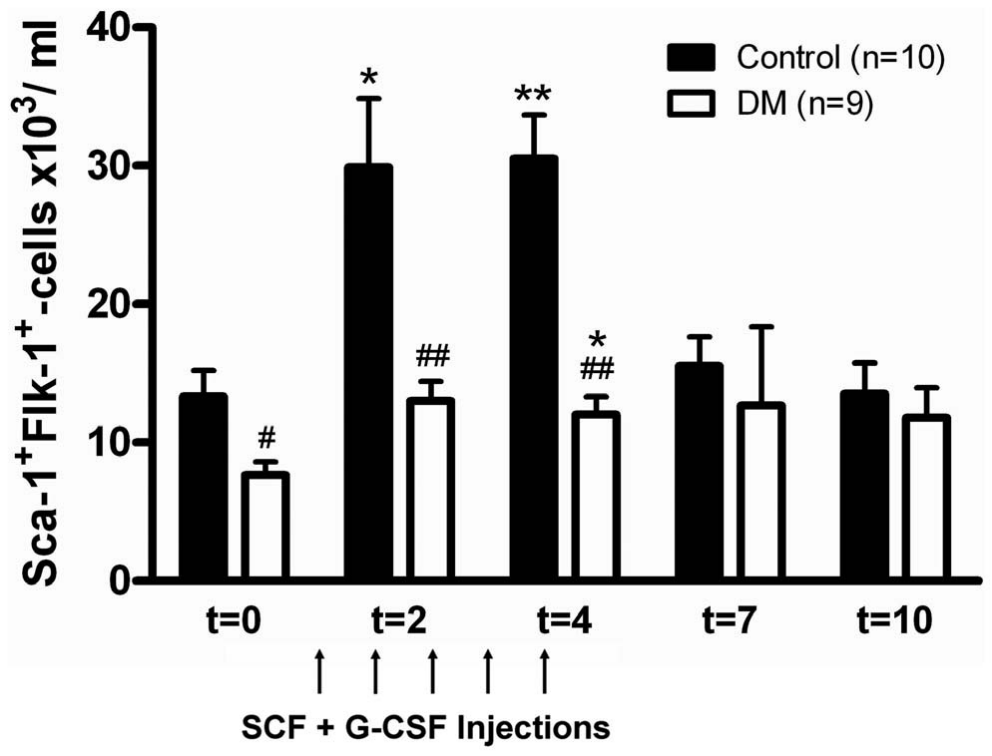
EPC levels and mobilization in diabetes. Diabetic animals have reduced levels of Sca1^+^Flk-1^+^ EPC in peripheral blood under steady-state (baseline) conditions compared to controls. After daily injection with mobilizing cytokines G-CSF and SCF from day 0 to 4, a significant mobilization of EPC was observed. EPC levels returned to baseline after cessation of cytokine injection. In contrast, diabetic animals showed a diminished mobilization response. P<0.001 for interaction of time and diabetes in 2-way ANOVA. ^#^P<0.05 compared to controls, ^##^ P<0.01 compared to controls, *P<0.05 compared to baseline, ** P<0.01 compared to baseline

Despite the normal bone marrow progenitor cell levels observed in diabetic mice peripheral blood Sca-1^+^Flk-1^+^ EPC were lower in diabetic mice during steady state conditions (diabetic, 7.7±0.9; control, 13.3.±1.9×10^3^/ml; P = 0.020; Fig. 1). Upon the mobilizing stimulus with G-CSF and SCF, a robust mobilization response was observed in control mice for all progenitor cell subtypes. Sca-1^+^Flk-1^+^ EPC levels reached 30.5±3.2×10^3^ EPC per ml of blood after maximal stimulation at day 4, corresponding to a 129% increase of 17.2±3.0×10^3^ EPC per ml (P = 0.003; Fig. 1). During the injection phase from day 0 to 4 EPC remained high and returned to baseline levels thereafter. In contrast, in diabetic mice EPC levels increased only moderately, although significant, with 4.3±0.8×10^3^ EPC per ml in response to the G-CSF/SCF injections to reach values of 12.0±1.3×10^3^ EPC per ml blood, corresponding to an increase of 56% compared to baseline levels (P = 0.009). Hence EPC levels differed significantly between diabetic and non-diabetic animals after maximal stimulation at day 4 (P<0.001). Additionally, a similar pattern of lower steady state progenitor cell levels and a blunted mobilization in response to G-CSF and SCF was observed for both the Sca-1^+^ and c-Kit^+^ HPC in diabetic mice. If progenitor cell populations were expressed relative to the total number of WBC the differences were in general less pronounced (Table 1).

#### Diabetic bone marrow fails to recover after 5-FU challenge

**Figure 2:**
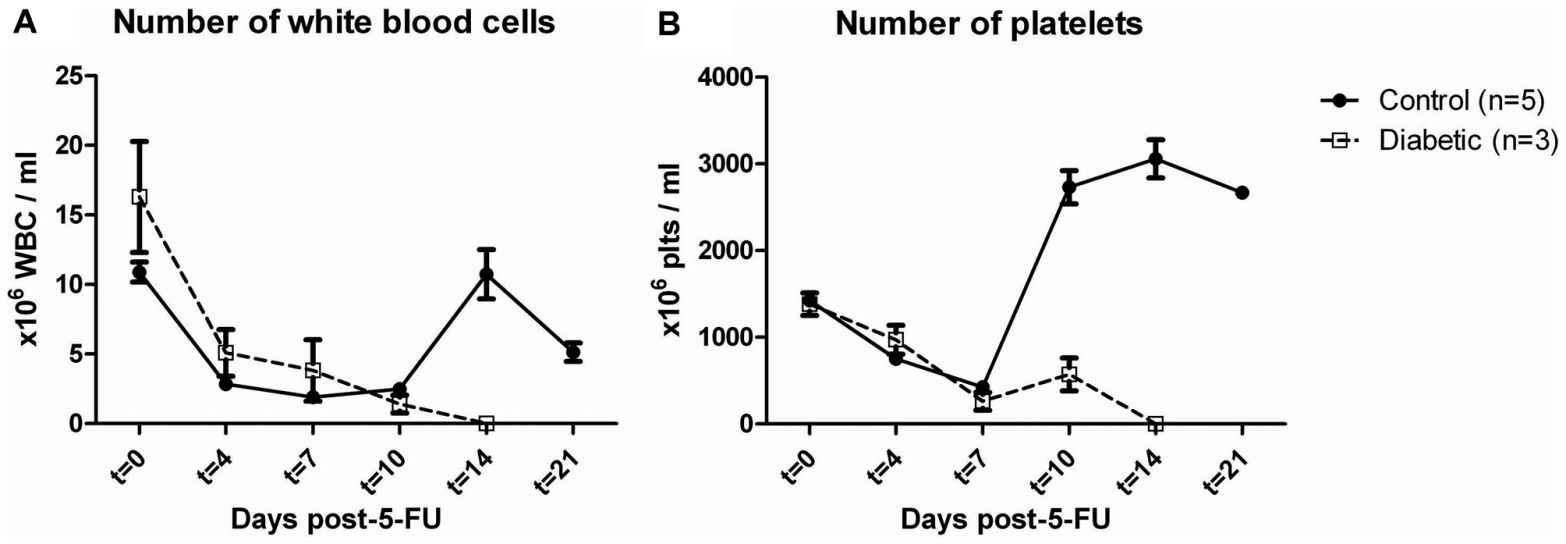
Diabetic mice do not recover after a 5-FU challenge. After 5-FU injection, a reduction in both WBC (**A**) and platelets (**B**) was observed in control and diabetic animals, which was maximal at 7 days after injection. Control animals fully recovered with restoration of WBC levels and platelets, displaying a typical rebound thrombocytosis after recovery. In contrast, diabetic animals died between day 10 and 14 with severe peripheral blood leukopenia and thrombopenia.

Diabetic and control animals were injected with 5-FU as an *in vivo* model for hemangiogenic and vascular niche recovery [18]. 5-FU injection causes destruction of proliferating bone marrow progenitor cells and sinusoidal endothelial cells, without affecting quiescent cells, and leads to a depression in peripheral blood WBC and platelet numbers. Recovery depends on the presence and function of quiescent multipotent stem cells, but particularly on functional vascular niche regeneration as a result of bone marrow neoangiogenesis [18], [36]. After 5-FU injection we observed a reduction in both WBC and platelets that was most pronounced 7 days after injection (Fig. 2A and B). As expected, control animals fully recovered with restoration of WBC and platelet levels, displaying a typical rebound thrombocytosis after recovery. In contrast, all diabetic animals died between day 10 and 14 due to a failure to repopulate with severe leukopenia and thrombopenia (Fig. 2A and B).

#### Diabetes and hyperglycemia impair the HPC supportive function of bone marrow stroma

**Figure 3:**
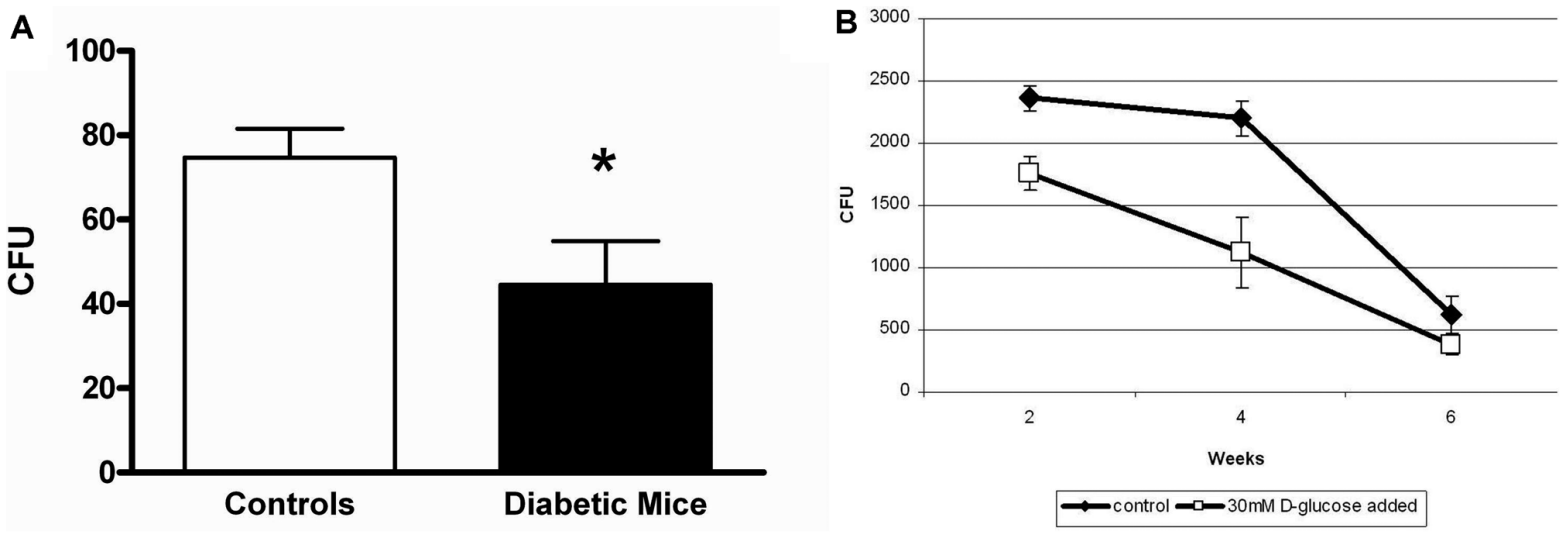
Progenitor cell support by bone marrow stroma ex vivo or endothelial cells in vitro. Plastic-adherent bone marrow stromal cells were grown to confluence from isolated bone marrow cell suspensions. Stromal layers from control (n = 10) and diabetic (n = 8) mice had a comparable morphology and growth pattern. Stromal layers were then used as feeder layer for human CD34^+^ HPC. (**A**)The number of CFU derived from 10-day co-cultures was significantly lower for diabetic stroma than for control stroma. Human CD34^+^ HPC were co-cultured with E4Orf1-transfected HUVEC for six weeks. At several time points, hematopoietic colonies (CFU) were counted. (**B**) Fewer CFU were obtained from CD34^+^ HPC co-cultured on endothelium in the presence of hyperglycemia than under control conditions. * P<0.05.

Plastic-adherent bone marrow stromal cells, a heterogeneous cell population including fibroblasts, endothelial cells, osteoblasts and potentially various other cell types, from diabetic and control primary cultures of mouse bone marrow cell suspensions were grown to confluence to serve as feeder layers for co-cultured human CD34^+^ CB-HPC. Stromal cell morphology and growth pattern was similar between cultures from diabetic and control animals (data not shown). However, the number of CFU cells derived from 10-day co-cultures was significantly lower for diabetic stroma compared to control stroma (45±10 vs. 75±7 CFU/well; P = 0.023, Fig. 3A). The distribution over the various types of CFU colonies was not affected (data not shown). Of note, cells were cultured using identical medium with a standard D-glucose concentration. As NO is essential for progenitor cell mobilization and maintenance we assessed the protein levels of the NO-producing enzyme eNOS by ELISA, which appeared to be significantly reduced in diabetic stroma (diabetic, 1.9±0.4; control, 4.1±0.4 AU; P = 0.0055). Quantitative real-time PCR for eNOS mRNA showed a 3.9 fold reduced expression in diabetic stroma (P<0.01), suggesting that the reduced eNOS protein levels were due to decreased transcription of eNOS or enhanced posttranscriptional mRNA degradation.

Because diabetes is associated with endothelial dysfunction and sinusoidal endothelium is important for progenitor cell support and egress, we studied whether the observed impairment of stromal cells to support progenitor cells under diabetic conditions might also be reflected in endothelial cells in an established *in vitro* model for the ‘vascular niche’. For this purpose we co-cultured human CD34^+^ CB-HPC with E4Orf1-transfected HUVEC in regular versus hyperglycemic conditions. Indeed, CD34^+^ CB-HPC cultured on this vascular niche model in the presence of hyperglycemia generated less CFU-generating cells compared to normoglycemic conditions (Fig. 3B). The distribution over the various types of CFU was not affected by hyperglycemia. We did not observe any direct effects of hyperglycemia on CD34^+^ CB-HPC survival or migratory function when these cells were kept in suspension overnight in the absence of endothelium (data not shown).

## Discussion

Chronically reduced EPC levels that are relatively unresponsive to mobilizing cues may hamper maintenance and regeneration of the vascular endothelium and impair neovascularization in response to ischemia in diabetes. In this study we show impaired mobilization of endothelial and hematopoietic progenitor cells in diabetes, despite undisturbed bone marrow levels. Furthermore, diabetic bone marrow was unable to repopulate after 5-FU administration, a model for hemangiogenic bone marrow regeneration. Moreover, *in vitro* HPC supportive function of diabetic stroma and endothelium in a hyperglycemic environment was impaired, in conjunction with reduced stromal eNOS expression. Our *in vitro* and *in vivo* data suggest that even short-term diabetes impairs progenitor cell supportive and mobilizing capacity of the bone marrow, which might particularly result from ‘vascular niche’ dysfunction.

The reported amount of bone marrow stem cells in diabetes are discordant, some studies show normal primitive Lin^−^ Sca-1^+^ c-Kit^+^ (LSK) HPC levels [6], [37], whereas others report decreased [38] or even increased [30] quantities. These discordant results probably reflect temporal changes as an effect of diabetes duration. In our study bone marrow Sca-1^+^Flk-1^+^ EPC levels were undisturbed consistent with observations by others in early stage diabetes [30], [37], [39], indicating that the attenuated mobilization response observed in the present study is not due to reduced pools of progenitor cells present in the bone marrow. In line with this, the number of *ex vivo* cultured bone marrow EPC was unaffected. Impaired progenitor cell mobilization may underlie the chronically reduced EPC levels under steady state conditions as well as the blunted EPC mobilization in response to progenitor cell mobilizing cues observed in diabetes. In the present study exogenous administration of G-CSF and SCF was used to mobilize progenitor cells from the bone marrow to the circulation. G-CSF and SCF are amongst the cytokines that are endogenously released in response to an ischemic event [40] and have been shown to synergistically induce progenitor cell mobilization [33], [41]. In non-diabetic mice, a pronounced over 2-fold increase in circulating EPC levels was indeed observed, while this mobilization response after G-CSF/SCF-injections was significantly blunted in diabetic animals. This is in line with observations in animal and patient studies reporting less efficient progenitor cell mobilization in response to an acute ischemic event in diabetic compared to non-diabetic subjects [6], [29], [42]. Additionally, Ferraro et al. showed that diabetic patients that underwent G-CSF elicited HPC mobilization prior to autologous bone marrow transplantation for hematologic conditions were not able to mobilize CD34^+^ HPC as effective as non-diabetic patients [30]. Furthermore, Fadini et al. reported that dysfunctional progenitor cell mobilization in response to exogenous G-CSF in both type 1 and type 2 diabetic patients extended to a broad range of progenitor cell populations, CD34^+^ HPC and CD34^+^KDR^+^ EPC amongst others [24].

Several pathologic processes that occur in the bone marrow under diabetic circumstances, such as dysfunction of the bone marrow niches [30], altered gene expression [38], [39] and cytokine signaling [24], [38], and bone marrow vasculo- [43] and neuropathy [31], have been implicated to impair bone marrow function and progenitor cell differentiation and mobilization. Here we focused on the effects of diabetes on the progenitor cell supportive role of the stromal compartment of the bone marrow by studying the interplay between progenitor cells and supporting stromal cells in two *in vitro* models. First the progenitor cell supportive function of primary diabetic bone marrow stroma was assessed by co-culturing the plastic-adherent stromal bone marrow fractions from mice with human CD34^+^ CB-HPC. This co-culture model supported HPC to survive and give rise to CFU after an *ex vivo* culture period of 10 days. However, when bone marrow stromal cells from diabetic mice were used fewer CFU were obtained compared to stromal cells from non-diabetic mice, indicating that diabetes induces impairment in the progenitor cell supportive function of the bone marrow stroma. Interestingly, these observations were made under normoglycemic culture conditions, suggesting that the diabetic stromal cell impairment is – at least to some extent – imprinted upon the cells. Furthermore, we showed that eNOS expression was reduced in diabetic stroma, which could partially explain the reduced supportive function, since NO is an important factor in progenitor cell mobilization, proliferation and maintenance [44].

The second *in vitro* model particularly focused on the effects of hyperglycemia on a progenitor cell supporting endothelial environment resembling the ‘vascular niche’ of the bone marrow. Therefore isolated human endothelial cells expressing E4Orf1 were co-cultured with human CD34^+^ CB-HPC. E4Orf1 expression in primary endothelial cell cultures results in a capacity of the endothelial cells to remain in a confluent state for a substantially prolonged period of time and the capacity to provide a functional vascular niche for progenitor cell expansion [35]. The extended endothelial survival is mediated by chronic Akt-phosphorylation and FGF-2/FGF-R1 activation [35]. Using this model fewer CFU were obtained under hyperglycemic conditions compared to normoglycemic conditions. This observation suggests that the endothelial capacity to support progenitor cells is impaired under hyperglycemic conditions, which is possibly due to disturbed interaction between the progenitor and endothelial cells.

Diabetes is associated with systemic microangiopathy, which has been shown to extend to the bone marrow causing capillary rarefaction, increased microvascular permeability, and endothelial cell apoptosis and dysfunction [43]. To assess the function and regeneration of the bone marrow vasculature *in vivo*, both diabetic and non-diabetic animals were injected with 5-FU. 5-FU causes a destruction of the cycling hematopoietic cells as well as the bone marrow sinusoids, while preserving quiescent stem and vascular progenitor cells. The recovery after 5-FU induced myelosuppression depends on a mutual restoration of hematopoiesis and angiogenesis and is therefore of particular value to study conditions that affect hemangiogenic restoration of the bone marrow [18], [36]. Diabetic mice proved to be dramatically impaired in recovering from 5-FU challenge and died during the experiment from bone marrow failure while control animals all recovered. This failure of diabetic animals to recover from 5-FU challenge is consistent with dysfunctional hemangiogenic reconstitution due to impaired restoration of hematopoiesis, angiogenesis, or a combination hereof. We cannot rule out the fact that the hyperglycemic condition had an effect on the long-term repopulating stem cells in the bone marrow explaining reduced progenitor cell numbers and repopulating potential, however overnight exposure to hyperglycemia did not have any marked effects on CD34^+^ HPC *in vitro* suggesting that impaired restoration of the ‘vascular niche’ is the key factor in the dysfunctional bone marrow recovery after 5-FU in diabetes. However the lack of effects after overnight exposure of CD34^+^ HPC to hyperglycemia *in vitro*, cannot fully rule out effects after sustained *in vivo* exposure. Importantly, Orlandi et al. found no specific impairment of the ‘vascular niche’ in diabetic mice with 20 weeks duration of diabetes, however they focused on quantification of SLAM^+^-cells, a specific osteoblast type that is localized to the vascular niche, but did not study functionality of the ‘vascular niche’. Furthermore, Orlandi et al. found that the bone marrow content of LSK HPC in diabetic mice is unaffected for up to 12 weeks duration of diabetes such as in our study, although a decline in number and engraftment efficiency was observed in mice after prolonged (20 weeks) duration of diabetes [39]. It is therefore possible that the bone marrow progenitor cell content remains unaffected in the initial phase of diabetes, but that long-term diabetes leads to reduced bone marrow EPC levels [38], [39].

The present study aimed primarily at providing functional data on the interaction of bone marrow stromal and progenitor cells rather than complete mechanistic insight regarding the influence of diabetes on bone marrow function and progenitor cell mobilization. Further studies are required to identify the specific pathways involved in diabetes induced impairment of progenitor cell mobilization and the progenitor cell supportive role of bone marrow stroma and the bone marrow endothelium in particular. In the regulation of progenitor cell mobilization, various cytokines, proteases, and cell-cell contact proteins play a central role. A critical pathway involves MMP-9-mediated cleavage of membrane-bound kit-ligand, releasing soluble kit ligand or SCF, which triggers progenitor cells to transfer from a quiescent to a proliferative niche [45], [46]. The activation of MMP-9 has been shown to be NO-dependent [21]. Based on bone marrow transplantation experiments between eNOS knockout mice and wild-type controls, it was shown that EPC mobilization in response to VEGF specifically depends on NO produced in bone marrow stromal cells; and not the progenitor cells themselves [21]. As diabetes and hyperglycemia have been shown to attenuate endothelial NO-production, this may be involved in the impaired function of the bone marrow endothelium. Indeed, eNOS in diabetic bone marrow displays an aberrant enzyme function [47] and altered expression in bone marrow stroma, as shown in the current study, as well as in mononuclear cells [38] and increasing NO-availability by hyperoxia restored EPC levels in diabetic animals [28]. In addition, bone marrow oxidative stress levels are increased in diabetic animals [38], [43] and increasing antioxidant levels prevented the development of bone marrow microangiopathy [43]. Also, in a study of bone marrow plasma cytokine levels in diabetic mice, various factors including VEGF were reduced [39]. In this study, SDF-1α levels were reduced without reaching statistical significance. However, Tepper et al. found the modulation of SDF-1α bone marrow levels in diabetic mice after peripheral skin injury to be significantly disturbed leading to impaired EPC mobilization. Restoration of SDF-1α induced mobilization by administration of plerixafor restored attenuated EPC mobilization supporting a pivotal role of the SDF-1α bone marrow ‘mobilizing switch’ in diabetes [37]. Abnormality of the SDF-1α signaling pathway, in particular disturbed upregulation of CD26/DPP-4 in response to G-CSF, was shown to play a role in disturbed progenitor cell mobilization in humans with diabetes as well [24], [48],

In line with the observation in humans of Fadini and co-workers [24], we found that the impaired mobilization of progenitor cells in diabetic animals was not restricted to the EPC subfraction of the HPC, but extends to a broader range of progenitor cells. HPC transplantation forms a widely accepted and well-established treatment strategy for both benign and malignant hematologic conditions [49], and the novel applications of HPC transplantation or transplantation of its specific subpopulations in for instance autoimmune and cardiovascular disease have been appreciated more recently [49], [50]. A common characteristic of these strategies is that their success–at least partly–depends on the number of CD34^+^ progenitor cells that can be isolated from the blood or bone marrow [51], [52]. It has been recently shown that diabetic patients undergoing HPC mobilization with G-CSF for hematologic conditions are ‘poor mobilizers’ of CD34^+^ HPC [30], which underlines that the results of the present study are also relevant beyond the cardiovascular field.

This study has some limitations. First, since this study particularly focused on the mobilization of EPC we primarily characterized these Sca-1^+^Flk-1^+^ cells. In order to study the influence of diabetes on a broader range of progenitor cells and to exclude an exclusive effect on EPC we additionally studied cells that were either Sca-1^+^ or c-Kit^+^. The veritable murine hematopoietic stem cells are Lin^−^ Sca-1^+^c-Kit^+^ cells, even if it would be expected that mobilization of these cells is disturbed in our animal model, we cannot draw conclusions on this specific progenitor cell population. Second, *in vitro* experiments were performed using CD34^+^ CB-HPC, instead of bone marrow CD34^+^ cells. The advantage of cord blood derived CD34^+^cells is that they are relatively free of potential confounding ‘environmental challenges’, such as aging and disease, which could negatively affect cell function. Furthermore, we performed interspecies experiments, which could have potentially influenced the results of the individual experiments. However, such interspecies experiments have been performed previously, showing that mouse-derived stromal cells could effectively support human progenitor cells [53], [54]. Additionally, cord blood cells have a relatively low immunogeneity compared to bone marrow cells [55], [56], which could have reduced the effects of interspecies differences. Moreover, these factors were all similar in the diabetic as well as in the non-diabetic experiments and therefore unlikely to have resulted in bias.

In conclusion, early stage experimental diabetes results in reduced steady state EPC levels and a blunted mobilization response, despite undisturbed progenitor cell levels in the bone marrow. Furthermore, we show that hemangiogenic regenerative potential of diabetic bone marrow is impaired. Moreover, diabetic stroma, wherein eNOS expression is reduced, is less effective in its HPC supporting function *in vitro*, which could be a result of ‘vascular niche’ dysfunction in particular. Therapeutic interventions known to improve endothelial function, such as enhanced NO bioavailability, may also have beneficial effects on the endothelium residing in the vascular niche, hence partly restoring the dysfunctional EPC mobilization in diabetes.
